# Dosing *de novo* combinations of two targeted drugs: Towards a customized precision medicine approach to advanced cancers

**DOI:** 10.18632/oncotarget.7023

**Published:** 2016-01-25

**Authors:** Sariah Liu, Mina Nikanjam, Razelle Kurzrock

**Affiliations:** ^1^ Department of Hematology-Oncology, Kaiser Permanente San Diego Medical Center, San Diego, CA, USA; ^2^ Division of Hematology-Oncology, University of California Los Angeles, Los Angeles, CA, USA; ^3^ Center for Personalized Cancer Therapy and Division of Hematology and Oncology, UC San Diego Moores Cancer Center, San Diego, CA, USA

**Keywords:** oncology, targeted therapy, maximum tolerated dose, recommended phase 2 dose, precision medicine

## Abstract

Metastatic cancers harbor complex genomic alterations. Thus, monotherapies are often suboptimal. Individualized combinations are needed in order to attenuate resistance. To help inform selection of safe starting doses for novel, two-agent, targeted drug combinations, we identified clinical trials in adult oncology patients who received targeted drug doublets (PubMed, January 1, 2010 through December 31, 2013). The dose percentage was calculated for each drug: (safe dose in combination divided by single agent full dose) X 100. Additive dose percentage represented the sum of the dose percentage for each drug. A total of 144 studies (N = 8568 patients; 95 combinations) were analyzed. In 51% of trials, each of the two drugs could be administered at 100% of their full dose. The lowest safe additive dose percentage was 60% if targets and/or class of drugs overlapped, or in the presence of mTor inhibitors, which sometimes compromised the combination dose. If neither class nor target overlapped and if mTor inhibitors were absent, the lowest safe additive dose percentage was 143%. The current observations contribute to the knowledge base that informs safe starting doses for new combinations of targeted drugs in the context of clinical trials or practice, hence facilitating customized combination therapies.

## INTRODUCTION

A rapidly growing body of knowledge in cancer genomics has unveiled a complicated and heterogeneous molecular landscape in metastatic cancers. Indeed, it has been recently reported that patients with advanced tumors interrogated by next generation sequencing often have unique and complex genomic profiles [1]. For instance, in 57 patients with metastatic breast cancer, 216 somatic aberrations in 70 different genes, including 131 distinct aberrations were observed. Furthermore, no two patients shared the same molecular portfolio [2]. Molecular heterogeneity exists between histologies as well as within the same diagnostic group, and even within individual patients [3]. This diversified genomic landscape speaks to the need for customized combination treatments based on the genetic signature associated with each tumor [4, 5].

Combination therapies with targeted agents are frequently adopted to overcome resistance and maximize efficacy. This is of particular importance given that patients with advanced cancer frequently carry multiple genomic aberrations simultaneously. In a patient-centric approach, combined therapies would be highly individualized, which poses challenges as to how to ensure safe delivery of *de novo* combined therapies. Phase I oncology trials are traditionally designed to address concerns about drug safety. However, with at least 300 anti-cancer drugs approved or in advanced clinical trials, there are about 45,000 two-drug combinations and approximately 4,500,000 three-drug combinations, with even higher numbers of combinations if all permutations of drug dosing are considered. Testing each combination therefore poses a herculean challenge. Furthermore, the most reasonable starting doses for clinical trials with two targeted agents remains unclear.

Outside the cancer field, drugs are combined routinely and safely, based on established algorithms, for patients with multiple comorbidities. Indeed, the average patient suffering from cancer is often on many therapeutic agents, often designated “polypharmacy,” for conditions as diverse as depression, heart disease, pain, and other illnesses. The safety of these drugs in combination has rarely if ever been formally tested in phase I studies. Yet physicians routinely prescribe a median of eight medications for patients with cancer, based on an understanding of drug-drug interactions and other factors [6]. Therefore, the prohibition against *de novo* combinations of drugs, and the demand for formal phase I testing of new combinations, often with slow and conservative dose escalation schemes, seems to be unique to the oncology sphere, and is perhaps a legacy from the era of cytotoxic drugs, which are toxic and have narrow therapeutic windows, especially compared to targeted agents that are generally better tolerated [7]. Importantly, within the context of oncology clinical trials, there is often considerable uncertainty as to what the initial dose levels should be, in the quest to balance safety, efficacy, and efficiency. In order to explore the correlation between dosing and toxicity for *de novo* combinations of targeted agents, we conducted an analysis of previously published clinical trials. The goal of this study was to use literature review to establish a process that would help determine safe initial dosing for novel combinations of two-drug combinations of targeted agents, in order to inform both clinical trials and practice.

## RESULTS

**Figure 1:**
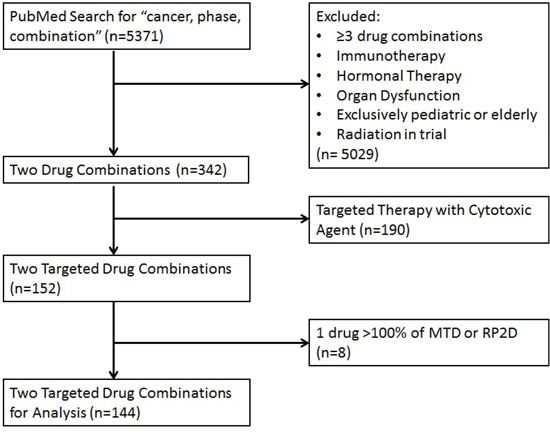
Consort Diagram Articles were identified by PubMed search and screened to identify two targeted drug combinations excluding studies of immunotherapy, hormonal therapy, radiation, or special populations (organ dysfunction, pediatric, or elderly patients).

**Figure 2:**
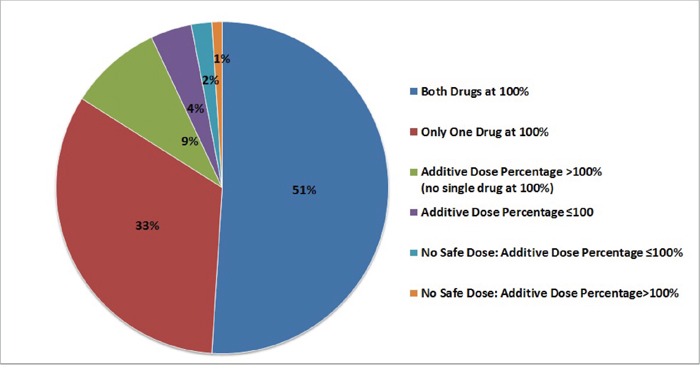
Graphical summary of additive dose percentages for two targeted therapies In 51% of studies both drugs could be administered at 100% of the FDA-approved dose/RP2D/MTD. In only 2% of studies (N = 3 studies) was the additive dose percentage tried ≤100% and no safe dose found. For the bevacizumab and sorafenib combination, other studies have found acceptable safety at 50% and 25% of the dose [22] or for 50% and 50% of the dose [30]. The only combination with undefined safety dosing in this group (and no alternative studies demonstrating safety) was the combination of pazopanib and temsirolimus [27]; dose-limiting toxicity in this trial included fatigue, and did not include acute irreversible events.

During the four-year period of publications evaluated, the total number of trials of two targeted agents that met the inclusion criteria was 144 (8568 patients; 95 drug combinations) (**Supplemental Table 1, Figure 1, Figure 2, Table 1**). A dose percentage was calculated to compare the dose of drug used in each combination to the single agent recommended dose (prioritizing the Food and Drug Administration- (FDA-) approved dose or, if not FDA approved, the recommended phase 2 dose (RP2D) or maximum tolerated dose (MTD), respectively) and the sum of the dose percentages for the combination was referred to as the additive dose percentage (see Methods, paragraph on Calculating “dose percentage”).

**Table 1:** Two targeted drug combinations reported over four years (Phase I, II, III studies on PubMed January 1, 2010 to December 21, 2013)

	Two targeted agents^a^
Number of trials	144
Number of drug combinations	95
Number of patients	8568
Median (range) additive dose percentage	200% (60%-200%)
Number (percent) of trials where ≥ one drug dose percentage was 100%	121 (84%)
Number of drug combinations where ≥ one drug dose percentage was 100%	78
Number of patients where ≥ one drug dose percentage was 100%	7748
Median (range) percentile for second drug when one drug dose percentage was 100%	100% (25%-100%)
Number (percent) of trials where each drug's dose percentage was 100% (e.g. additive dose percentage^b^ = 200%)	74 (51%)
Number of drug combinations where each drug's dose percentage was 100% (e.g. additive dose percentage = 200%)	48
Number of patients where each drug's dose percentage was 100% (e.g. additive dose percentage = 200%)	5229
Number (percent) of trials where additive dose percentage was >100%, with no single drug given at 100%	13 (9%)
In trials where additive dose percentage was >100%, with no single drug given at 100%, median (range) of additive dose percentage	127% (104%-148%)
Number (percent) of trials where additive dose percentage was ≤100% and safe dose was found	5 (4%)
In trials where additive dose percentage was ≤100% and safe dose was found, median (range) of additive dose percentage	75% (60%-100%)
Number (percent) of trials where additive dose percentage was ≤100% and safe dose was not found^c^	3 (2%)
In trials where additive dose percentage was ≤100% and safe dose was not found, median (range) of additive dose percentage studied	75% (65%-100%)
Number (percent) of trials that were aborted early with additive dose percentage >100%	2 (1%)
In trials that were aborted early with additive dose percentage >100%, median (range) of additive dose percentage	200%

a Excludes hormonal modulators and immunotherapy

b Additive dose percentage = (dose of drug A in combination/standard dose of drug A as a single agent) X 100 + (dose of drug B in combination/standard dose of drug B as a single agent) X 100.

c See Results for details. In the case of two trials in which safe dose could not be defined, other trials of the same combination did define a safe dose (additive dose percentage = 100% or 75%); one trial had fatigue as a dose-limiting toxicity, but no acute or irreversible toxicities.

### First drug at 100% dose percentage of the FDA-approved dose/RP2D/MTD

Between January 1, 2010 and December 31, 2013, 121 trials (including 78 drug combinations) were published (N = 7748 patients) where both agents were targeted and at least one was given at full (100%) dose [8–18] **(Supplemental references 1 to 110) (Table 2)**. These included 47 phase I trials (N = 1449 patients), 63 Phase II or III trials (N = 5742 patients), and 11 phase I/II combined trials (N = 557 patients). (In the phase I trials, only a subset of patients were treated at the RP2D/MTD)

**Table 2:** Summary of Two Targeted Drugs in Combination^a^

	Second drug at 100% dose percentage of FDA-approved dose/RP2D/MTD	Lowest safe dose percentage of second drug if both are of the same class and/or have overlapping targets	Lowest additive dose percentage of the combination
First drug at 100% dose percentage of FDA-approved dose/RP2D/MTD^b^	61% of trials(74/121)(Note: 74 of the 144 total trials (51%) administered each drug at 100% dose)	25% of FDA/RP2D/MTD^c^	125%
First drug at 51-99% dose percentage of the FDA-approved dose/RP2D/MTD	Not applicable(13 total trials)	29% of FDA/RP2D/MTD^d^	104%^d^ 143% (for non-overlapping targets and different classes)^e^
First drug at ≤ 50% dose percentage of the FDA-approved dose/RP2D/MTD	Not applicable(5 total trials)	10% of FDA/RP2D/MTD^f^	60% (overlapping targets in each case)^f^ 90% (for non-overlapping targets, but both same class (small molecule inhibitors)^g^

a The five studies where no safe dose was found or study was aborted early due to unacceptable toxicity were excluded from this table and include: bevacizumab and sorafenib [28, 29], pazopanib and temsirolimus [27], bevacizumab and everolimus [31], and bevacizumab and temsirolimus [32]

b First drug had the dose percentage closest to the FDA-approved/RP2D/MTD dose

c In these cases, the combinations were same class (small molecule inhibitors) with non-overlapping targets (sorafenib at 100% with everolimus at 25%, and imatinib at 100% with everolimus at 25%) [12–14].

d Sunitinib was at 75% and everolimus at 29% [20]

e Rapamycin was at 93% and bevacizumab was at 50% [19]

f Bevacizumab with vatalinib [26] and bevacizumab with telatinib [25] each included an anti-VEGF antibody and a small molecule VEGFR inhibitor (both at 10% and 50%, respectively)

g Sorafenib was at 50% and temsirolimus at 40% [23]; however the combination of pazopanib and temsirolimus was above the FDA-approved/RP2D/MTD at an additive dose percentage of 65% (albeit with no acute or irreversible side effects and with the nonspecific side effect of fatigue as dose limiting in one patient).

Abbreviations: MTD = maximum tolerated dose; RP2D = recommended phase II dose

The median dose percentage for the second agent was at 100% of the FDA-approved dose/RP2D/MTD (range, 25% to 100%). The median (range) for the additive dose percentage was 200% (125% to 200%) of the additive FDA-approved dose/RP2D/MTD. The lowest safe additive dose percentage was 125%; this lower dose was needed in some studies where both drugs were of the same class, i.e., small molecule inhibitors.

In total, 75 trials (51% of the 144 trials of the trials) (N = 48 drug combinations) administered each targeted agent at 100% dose percentage (N = 5229 patients received each drug at 100% dose percentage). These trials (wherein additive dose percentage was 200%) included the following types of combinations: 10 combinations that involved an antibody and small molecule tyrosine kinase inhibitor (TKI); 7 combinations, two antibodies; 17 combinations, antibody and small molecule non-TKI; 6 combinations, two small molecule TKIs; 6 combinations, a small molecule non-TKI and small molecule TKI; and 2 combinations, involved two small molecule non-TKIs. In 3 of the 48 combinations where each drug was given at 100%, dose percentage, the target of the two molecules overlapped. These included the following: gefitinib and nimotuzumab (both targeting epidermal growth factor receptor (EGFR)) [9]; bevacizumab and ABT-510 (targeting vascular endothelial growth factor (VEGF)) [8]; and trastuzumab and pertuzumab (targeting HER2) [10, 11].

Subset analyses with two antibodies, two small molecule inhibitors, and an antibody with a small molecule inhibitor were performed. For all 9 studies of two antibodies given in combination, each drug was given at 100% of the FDA-approved dose/RP2D/MTD dose. When two small molecules were administered, each drug could be administered at 100% of the dose in 25 of the 68 total trials (37%) **(Table 3).** When a small molecule and antibody were administered in combination, each drug could be administered at 100% in 40 out of 55 total trials (73%) **(Table 3).** Limitations of this analysis are due to the small number of antibody-antibody combinations (N = 9).

**Table 3:** Summary of Subset Analysis for Combination of Two Small Molecule Inhibitors, as well as Small Molecule Inhibitor and Antibody Combinations^a^

**Combination of Two Small Molecule Inhibitors (N = 68 Studies)**		
	Second drug at 100% dose percentage of FDA-approved dose/RP2D/MTD	Lowest additive dose percentage of the combination
First drug at 100% dose percentage of FDA-approved dose/RP2D/MTD	44% of trials(25/57)(Note: 25 of the 68 total trials (37%) administered each drug at 100% dose)	125%^b^
First drug at 51-99% dose percentage of the FDA-approved dose/RP2D/MTD	Not applicable(9 total trials)	104%^c^
First drug at ≤ 50% dose percentage of the FDA-approved dose/RP2D/MTD	Not applicable(2 total trials)	90%^d^
**Combination of Small Molecule Inhibitor and Antibody Combinations (N = 62 Studies)**		
First drug at 100% dose percentage of FDA-approved dose/RP2D/MTD	73% of trials(40/55)(Note: 40 of the 62 total trials (65%) administered each drug at 100% dose)	150%^e^
First drug at 51-99% dose percentage of the FDA-approved dose/RP2D/MTD	Not applicable(4 total trials)	117%^f^ 143% (for non-overlapping targets)^g^
First drug at ≤ 50% dose percentage of the FDA-approved dose/RP2D/MTD	Not applicable(3 total trials)	60% (overlapping targets in each case)^h^

a All combinations of two antibodies had each drug given at 100% of the FDA-approved/RP2D/MTD dose

b In these cases, the combinations had non-overlapping targets (sorafenib at 100% with everolimus at 25%, and imatinib at 100% with everolimus at 25%) [12–14].

c Sunitinib was at 75% and everolimus at 29% [20]

d Sorafenib was at 50% and temsirolimus at 40% [23].

e Bevacizumab and erlotinib were each given at 50% and 100%, respectively [15–17] while panobinostat and bevacizumab were given at 50% and 100%, respectively [18].

f Vandetanib was at 67% and bevacizumab was at 50% [21].

g Rapamycin was at 93% and bevacizumab was at 50% [19].

h Bevacizumab with vatalinib [26] and bevacizumab with telatinib [25] each included an anti-VEGF antibody and a small molecule VEGFR inhibitor (both at 10% and 50%, respectively).

### First drug at >50% but < 100% dose percentage of the FDA-approved dose/RP2D/MTD

There were 13 trials (N = 13 drug combinations) where the first drug was administered at >50% but < 100% of the FDA-approved dose/RP2D/MTD due to toxicity of higher doses [19–21] **(Supplemental references 111-120)**. The lowest safe additive dose of the combination was 104% and the latter was required for sunitinb (75% of dose) combined with everolimus (29% of dose) [20]. The lowest safe additive dose percentage was 143% [19] (rapamycin and bevacizumab) when the drugs did not overlap in either class or target (**Table 2**)

### First drug at less than or equal to 50% dose percentage of the FDA-approved dose/RP2D/MTD

Five trials (bevacizumab and sorafenib [22]; sorafenib and temsirolimus [23]; sorafenib and sirolimus [24]; bevacizumab and telatinib (VEGFR inhibitor) [25]; bevacizumab and vatalinib (VEGFR inhibitor) [26] were published, where both agents were targeted and the first drug was administered at ≤ 50% dose percentage of the FDA-approved dose/RP2D/MTD due to toxicity of higher doses, thus the additive dose percentage was **≤** 100% of the additive FDA-approved dose/RP2D/MTD. The dose percentage for bevacizumab and sorafenib was 50% and 25% of the RP2D of each drug, respectively; for sorafenib and temsirolimus, 50% and 40%, respectively; for sorafenib and sirolimus, 50% and 50%, respectively; for bevacizumab and telatinib, 10% and 50%, respectively; for bevacizumab and vatalinib, 10% and 50%, respectively (additive dose percentage = 75%, 90%, 100%, 60% and 60%). Of note, the lowest additive dose percentages (75%, 60% and 60%, respectively) applied to bevacizumab and sorafenib, bevacizumab and telatinib, and bevacizumab and vatalinib, which, in each case, overlap in their targeting angiogenesis. In addition, the combination of pazopanib and temsirolimus was given at 65% additive dose percentage and was considered above the MTD, but the toxicity was fatigue, which is often hard to quantify [27] **(Table 2).**

### mTor inhibitor-based combinations

Combinations with mTor inhibitors such as everolimus or temsirolimus often required compromised doses: (i) the combination of sorafenib (100% of dose) with everolimus necessitated dosing the latter at 25% [12]; (ii) the everolimus dose was reduced to 25% in combination with imatinib, when used at 100% [13, 14]; (iii) when sunitinib (75% of dose) was combined with everolimus, only 29% of the dose of the latter could be given [20]; and (iv) as mentioned above, the dose of pazopanib (25%) and temsirolimus (40%) resulted in dose limiting toxicity of fatigue [27].

### Two targeted agents where the additive dose percentage was ≤ 100% and safety was unacceptable

Three trials (bevacizumab and sorafenib (2 trials giving 50% and 50%; 50% and 25%) [28, 29]; one trial, pazopanib and temsirolimus (25% and 40% dose, respectively) [27]) were published where the lowest dose level did not have an acceptable safety profile. Of interest, for the bevacizumab and sorafenib combination, other studies have found acceptable safety at 50% and 25% of the dose [22] or for 50% and 50% of the dose [30]. Therefore the only combination with an undefined safety dosing in this group (and no alternative studies demonstrating safety) was the study of pazopanib and temsirolimus mentioned above [27]. As mentioned, dose-limiting toxicity in this study included fatigue, and did not include acute irreversible events.

### Two targeted agents where the study was aborted early or safety defined as unacceptable and the additive dose percentages investigated were > 100%

There were two trials published where the additive dose percentage was ≥ 100% and the studies did not find an acceptable dose: bevacizumab and everolimus (both drugs at 100%) [31]; bevacizumab and temsirolimus (both at 100% of each) [32]. These trials did not attempt to lower the dose.

## DISCUSSION

Targeted agents matched to advanced tumors bearing cognate alterations are often given as monotherapy. While significant salutary effects can be achieved [5, 33, 34] responses generally last only a few months. This is perhaps not surprising since metastatic malignancies mostly harbor multiple genomic alterations [35–38], strongly suggesting that individualized combination treatment will be need to be deployed to further improve outcomes.

When two targeted agents are combined, safety considerations may include whether both belong to the same class of drugs (e.g., both are small molecule inhibitors) or if there are overlapping targets (e.g. both target angiogenesis) [7]. In the current study, we have reviewed phase I-III clinical trials of targeted therapeutics over a four-year span (N = 8568 patients) to determine safe starting doses for novel two-drug combinations of targeted agents.

In over half of the studies, both drugs could be given at 100% of the individually defined optimum dose (i.e., the FDA-approved dose or RP2D or MTD) (**Table 2**). When giving full doses was not possible, studies were able to define safe starting doses by lowering the additive dose percentage of the combination. When one drug was given at 100% of the full dose, the lowest safe dose for the second drug was 25%. Lowering the dose was needed in the presence of two drugs of the same class when both were small molecule inhibitors or two drugs with overlapping targets. In some cases, neither drug was administered at 100% of the full dose. The lowest additive dose percentage was 60% and was relevant to bevacizumab and telatinib, and bevacizumab and vatalinib, which overlapped in their target of angiogenesis [25, 26]. However, other studies were able to administer 100% of each agent (200% additive dose percentage) despite overlapping targets: gefitinib and nimotuzumab (targeting EGFR) [9]; bevacizumab and ABT-510 (targeting VEGF) [8]; and trastuzumab and pertuzumab (targeting HER2) [10, 11]. Thus, the presence of overlapping targets needs to be considered for starting doses, but, in many cases, will not limit the ability to administer full doses of agents. Combinations that included mTor inhibitors such as everolimus or temsirolimus also resulted in compromised doses. At times, these combinations could not be given at more than 65 to 100% of the additive dose percentage. Finally, the lowest safe additive dose percentage for drugs with non-overlapping targets or class was 143%.

In implementing novel drug combinations tailored to the genomic aberration of each individual cancer, considerations include efficacy of the combination and toxicity. The effect of administration of less than 100% of the single agent MTD for combination therapies was addressed in two separate studies of phase I data. Jain et al demonstrated, in a single institution study of 24 clinical trials, that patients who received lower drug doses did not fare worse than those on higher doses, and suggested that targeted agents may have different dose response relationships than cytotoxic chemotherapies [39]. A separate study of 55 clinical trials sponsored by a single entity with multiple sites suggested that patients on higher doses had better response rates and overall survival [40]. Thus, it is unclear if dose reductions to allow for the administration of multiple agents will alter efficacy.

This study has several limitations. First, the publications reviewed for the current analysis were limited to two-drug combinations (targeted agents) in adult patients without organ dysfunction. The results are likely not applicable to patients with renal or hepatic impairment, or children, who may require dosing modifications depending on metabolism of the therapeutic or maturation, respectively, and were excluded from the current analysis as often occurs in clinical trials. In addition, immunotherapies, hormonal modulators, and cytotoxics were not included, which may alter the additive dose percentages seen in the study as hormonal modulators and immunotherapies may be better tolerated in combination therapy while cytotoxics may increase toxicity. It is also plausible that some trials with two targeted agents that showed significant toxicity were never published, and so the data presented herein, while derived from a large number of patients, does not guarantee safety with all possible drug combinations. Drug-drug interactions and effects on metabolic proteins such as CYP enzymes, which can lead to changes in the pharmacokinetic profile of therapeutics, may have resulted in lower safe dose levels for combination therapy as compared to single agent dosing. While this was not addressed in the study, we still observed that, in 51% of trials, both drugs could still be administered at 100% of their FDA-approved dose/RP2D/MTD. The study also did not address target engagement for therapy where optimal doses may be lower than the FDA-approved dose/RP2D/MTD. Still it is of interest that for some drugs such as everolimus, administering 5 mg (which is 50% of the approved dose) offers 100% target engagement [41]. Another limitation of the study was that it included different types of trials (phase I, phase II, phase III trials) with different primary objectives. Finally, there are myriad of possible dosing schemes and some investigators or practitioners may want to hold one of the drugs at a preconceived dosing level. While our study did define that, if one drug is held at 100%, the lowest safe additive dosing percentage was 125%, it was not possible to define all permutations because they do not exist in the literature reviewed.

In conclusion, classical cytotoxic chemotherapy dosing was previously limited by significant toxicity, and thus the administration of two or more drugs in combination often necessitated very conservative initial dosing, even within the controlled environment of a clinical trial. Although targeted therapies can have fewer side effects than traditional cytotoxic chemotherapy, a process to calculate initial safe doses, either within a clinical trial or in practice, for combinations of two targeted agents, remains a matter of debate. The molecular heterogeneity of cancer indicates that prosecuting malignancies with an optimized personalized/precision medicine strategy will require combination therapy matched to individual molecular profiles. Yet, with an increasing number of targeted agents deployed in the clinic, there are thousands of drug combinations possible, and there is an increasingly urgent need for more knowledge that can inform safely combining them. Outside of oncology, for patients with multiple comorbidities, drugs are routinely given together based on established algorithms; indeed, the average cancer patient is on 5 to 10 drugs for their other health problems, often before starting treatment for their malignancy. The current study documented the following in adults with intact organ function treated with two targeted agents: (i) compromised dosing most often was needed for overlap of drug class (e.g., two small molecule inhibitors (but not two antibodies)) and/or targets (especially angiogenesis) or in the presence of mTor inhibitors; (ii) without overlap of class or target and in the absence of mTor inhibitors, the lowest safe additive dose percentage was 143%; (iii) in the presence of overlapping class and/or targets and/or mTor inhibitors, the lowest safe additive dose percentage was about 60%; and (iv) dose escalation to full dose was possible with most two targeted drug combinations, since over half of these combinations were administered safely at 100% dose percentage of the FDA-approved/RP2D/MTD of each drug (additive dose percentage = 200%). Therefore starting doses of two targeted drugs in combination in a clinical trial or practice could be about 70% of each drug if there is no overlap in targets or class and no mTor inhibitor in the regimen, and about 30% of each drug with overlap of class and/or target or inclusion of an mTor inhibitor. If one drug was held at 100% of full dose, the lowest safe starting dose of the second drug was 25%, and this was required in the case of overlapping drug class/target. Since over half of the combinations could be given with both drugs at full dose, in the absence of significant toxicity, intra-patient dose escalation can occur, to allow for improved efficacy if needed. Of course, further adjustments may be needed, depending on co-morbidities, patient age, organ function, other concomitant medications, and consideration of absorption, distribution, metabolism and excretion (ADME) of individual drugs. Despite these limitations, our current observations can help inform the safe starting dose of *de novo* two targeted agent combinations, both in clinical trials and practice, as a step toward customization of therapy to the complex molecular landscape seen in patients with cancer.

## MATERIALS AND METHODS

To identify research articles for the analysis, we first conducted a search of PubMed for studies published between January 1, 2010 and December 31, 2013, using the search terms “cancer, phase, combination.” We then manually screened the resulting articles and included studies that meet the inclusion criteria: (i) phase I-III clinical trials; (ii) solid tumors or hematology malignancy; and (iii) two-drug combination therapy where both drugs were targeted agents. Targeted agents are generally cytostatic and broadly include antibodies that have a specific protein as their target or small molecule inhibitors with low nM IC50s (concentration that results in 50% inhibition of enzyme function) for the specific protein target. Exclusion criteria were as follows: (i) the dose of any drug in the combination was greater than 100% of the standard dose as a single agent; (ii) the dose of any drug in the combination was chosen to be low due to reasons other than toxicity, such as optimized activity at lower doses due to different biological impact; (iii) the study was performed on selected patient populations such as pediatric, elderly, or patients with organ dysfunction; and (iv) the study treatment regimen included radiation. Hormonal agents and immonotherapeutics were excluded.

### Data

Clinical data were manually extracted from each clinical trial. Data included drug names, targets of action (the target of small molecule inhibitors was felt to be relevant if it was impacted at an IC50 <250 nM [42]), drug type, status of Food and Drug Administration (FDA) approval, number of drugs in the combination, disease, number of participants, dose of each drug in the combination, recommended phase 2 dose (RP2D) or maximal tolerated dose (MTD) achieved in the study, dose limiting toxicities (DLTs), and grade ≥ 3 toxicities.

### Calculating “dose percentage”

Based on the extracted data, a “dose percentage” was calculated, which was defined as the dose of one drug in the combination, divided by the standard dose of the same drug used as a single agent, multiplied by 100 ((dose percentage = dose of drug in the combination/standard dose of drug as a single agent) X 100). Single agent dose was defined as the FDA approved dose, or for drugs that were not FDA approved, the RP2D or MTD dose from phase I studies. FDA approved dose was always prioritized as the reference full dose and RP2D was prioritized over MTD. For drugs where the standard single agent dosing could be variable, we defined the lower standard dose as the accepted dose. “Additive dose percentage” for combinations of two targeted agents was calculated by adding the dose percentage of each drug in a given combination. Hence, in combination therapy, if the maximum safe dose percentage of drug A was 50% of the FDA-approved dose/RP2D/MTD of drug A as a single agent, and the maximum safe dose percentage of drug B was 25% of the FDA-approved dose/RP2D/MTD of drug B as a single agent, the “additive dose percentage” of the combination was 75%. The maximum “additive dose percentage” for any two-drug combination is 200% (i.e., 100% of each drug). The “first drug” was defined as the drug with the highest dose percentage of the combination (i.e., the drug with the dose that was closest to the FDA-approved dose/RP2D/MTD).

## SUPPLEMENTARY FIGURES AND TABLES




