# Supplementary material for: Dosing de novo combinations of two targeted drugs: Towards a customized precision medicine approach to advanced cancers
Source: Oncotarget. 2016 Jan 25;7(10):11310–20. doi: 10.18632/oncotarget.7023 (PMC4905475; doi:10.18632/oncotarget.7023)
Supplement: Supplementary file 1 [file oncotarget-07-11310-s001.pdf]

SUPPLEMENTARY REFERENCES

1. Fishman MN, Srinivas S, Hauke RJ, Amato RJ, Esteves B, Cotreau MM, Strahs AL, Slichenmyer WJ, Bhargava P, and Kabbinavar FF. Phase Ib study of tivozanib (AV-951) in combination with temsirolimus in patients with renal cell carcinoma. Eur J Cancer. 2013; 49: 2841-2850.
2. Felip E, Ranson M, Cedres S, Dean E, Brewster M, Martinez P, McNally V, Ross G, and Galdermans D. A phase Ib, dose-finding study of erlotinib in combination with a fixed dose of pertuzumab in patients with advanced non-small-cell lung cancer. Clin Lung Cancer. 2012; 13: 432-441.
3. Ciunci CA, Perini RF, Avadhani AN, Kang HC, Sun W, Redlinger M, Harlacker K, Flaherty KT, Giantonio BJ, Rosen MA, Divgi CR, Song HK, Englander S, et al. Phase 1 and pharmacodynamic trial of everolimus in combination with cetuximab in patients with advanced cancer. Cancer. 2014; 120: 77-85.
4. Goldman JW, Laux I, Chai F, Savage RE, Ferrari D, Garmey EG, Just RG, and Rosen LS. Phase 1 dose-escalation trial evaluating the combination of the selective MET (mesenchymal-epithelial transition factor) inhibitor tivantinib (ARQ 197) plus erlotinib. Cancer. 2012; 118: 5903-5911.
5. Yamamoto N, Murakami H, Hayashi H, Fujisaka Y, Hirashima T, Takeda K, Satouchi M, Miyoshi K, Akinaga S, Takahashi T, and Nakagawa K. CYP2C19 genotype-based phase I studies of a c-Met inhibitor tivantinib in combination with erlotinib, in advanced/metastatic non-small cell lung cancer. Br J Cancer. 2013; 109: 2803-2809.
6. Nemunaitis J, Hochster HS, Lustgarten S, Rhodes R, Ebbinghaus S, Turner CD, Dodion PF, and Mita MM. A phase I trial of oral ridaforolimus (AP23573; MK-8669) in combination with bevacizumab for patients with advanced cancers. Clin Oncol (R Coll Radiol). 2013; 25: 336-342.
7. Jhaveri K, Miller K, Rosen L, Schneider B, Chap L, Hannah A, Zhong Z, Ma W, Hudis C, and Modi S. A phase I dose-escalation trial of trastuzumab and alvespimycin hydrochloride (KOS-1022; 17 DMAG) in the treatment of advanced solid tumors. Clin Cancer Res. 2012; 18: 5090-5098.
8. Brown JR, Messmer B, Werner L, Davids MS, Mikler E, Supko JG, Fisher DC, LaCasce AS, Armand P, Jacobsen E, Dalton V, Tesar B, Fernandes SM, et al. A phase I study of escalated dose subcutaneous alemtuzumab given weekly with rituximab in relapsed chronic lymphocytic leukemia/small lymphocytic lymphoma. Haematologica. 2013; 98: 964-970.
9. Hudis C, Swanton C, Janjigian YY, Lee R, Sutherland S, Lehman R, Chandarlapaty S, Hamilton N, Gajria D, Knowles J, Shah J, Shannon K, Tetteh E, et al. A phase 1 study evaluating the combination of an allosteric AKT inhibitor (MK-2206) and trastuzumab in patients with HER2-positive solid tumors. Breast Cancer Res. 2013; 15: R110.
10. de Jonge MJ, Hamberg P, Verweij J, Savage S, Suttle AB, Hodge J, Arumugham T, Pandite LN, and Hurwitz HI. Phase I and pharmacokinetic study of pazopanib and lapatinib combination therapy in patients with advanced solid tumors. Invest New Drugs. 2013; 31: 751-759.
11. Nghiemphu PL, Lai A, Green RM, Reardon DA, and Cloughesy T. A dose escalation trial for the combination of erlotinib and sirolimus for recurrent malignant gliomas. J Neurooncol. 2012; 110: 245-250.
12. Blumenschein GR, Jr., Reckamp K, Stephenson GJ, O'Rourke T, Gladish G, McGreivy J, Sun YN, Ye Y, Parson M, and Sandler A. Phase 1b study of motesanib, an oral angiogenesis inhibitor, in combination with carboplatin/paclitaxel and/or panitumumab for the treatment of advanced non-small cell lung cancer. Clin Cancer Res. 2010; 16: 279-290.
13. Rosen PJ, Sweeney CJ, Park DJ, Beaupre DM, Deng H, Leitch IM, Shubhakar P, Zhu M, Oliner KS, Anderson A, and Yee LK. A phase Ib study of AMG 102 in combination with bevacizumab or motesanib in patients with advanced solid tumors. Clin Cancer Res. 2010; 16: 2677-2687.
14. O'Mahar SE, Campbell TC, Hoang T, Seo S, Kim K, Larson MM, Marcotte SM, LoConte NK, and Traynor AM. Phase I study of sunitinib and erlotinib in advanced nonsquamous non-small cell lung cancer. J Thorac Oncol. 2011; 6: 951-953.
15. Argiris A, Feinstein TM, Wang L, Yang T, Agrawal S, Appleman LJ, Stoller RG, Grandis JR, and Egloff AM. Phase I and pharmacokinetic study of dasatinib and cetuximab in patients with advanced solid malignancies. Invest New Drugs. 2012; 30: 1575-1584.
16. Quintela-Fandino M, Le Tourneau C, Duran I, Chen EX, Wang L, Tsao M, Bandarchi-Chamkhaleh B, Pham NA, Do T, MacLean M, Nayyar R, Tusche MW, Metser U, et al. Phase I combination of sorafenib and erlotinib therapy in solid tumors: safety, pharmacokinetic, and pharmacodynamic evaluation from an expansion cohort. Mol Cancer Ther. 2010; 9: 751-760.
17. Dean E, Middleton MR, Pwint T, Swaisland H, Carmichael J, Goodege-Kunwar P, and Ranson M. Phase I study to assess the safety and tolerability of olaparib in combination with bevacizumab in patients with advanced solid tumours. Br J Cancer. 2012; 106: 468-474.
18. van Cruijsen H, Voest EE, Punt CJ, Hoekman K, Witteveen PO, Meijerink MR, Puchalski TA, Robertson J, Saunders O, Jurgensmeier JM, van Herpen CM, and Giaccone G. Phase I evaluation of cediranib, a selective VEGFR signalling inhibitor, in combination with gefitinib in patients with advanced tumours. Eur J Cancer. 2010; 46: 901-911.

19. Cohen EE, Sharma MR, Janisch L, Llobrera M, House L, Wu K, Ramirez J, Fleming GF, Stadler WM, and Ratain MJ. A phase I study of sirolimus and bevacizumab in patients with advanced malignancies. Eur J Cancer. 2011; 47: 1484-1489.
20. Quek R, Wang Q, Morgan JA, Shapiro GI, Butrynski JE, Ramaiya N, Huftalen T, Jederlinic N, Manola J, Wagner AJ, Demetri GD, and George S. Combination mTOR and IGF-1R inhibition: phase I trial of everolimus and figitumumab in patients with advanced sarcomas and other solid tumors. Clin Cancer Res. 2011; 17: 871-879.
21. Vaishampayan UN, Burger AM, Sausville EA, Heilbrun LK, Li J, Horiba MN, Egorin MJ, Ivy P, Pacey S, and Lorusso PM. Safety, efficacy, pharmacokinetics, and pharmacodynamics of the combination of sorafenib and tanespimycin. Clin Cancer Res. 2010; 16: 3795-3804.
22. Garrett CR, Siu LL, El-Khoueiry A, Buter J, Rocha-Lima CM, Marshall J, LoRusso P, Major P, Chemidlin J, Mokliatchouk O, Velasquez L, Hayes W, Feltquate D, et al. Phase I dose-escalation study to determine the safety, pharmacokinetics and pharmacodynamics of brivanib alaninate in combination with full-dose cetuximab in patients with advanced gastrointestinal malignancies who have failed prior therapy. Br J Cancer. 2011; 105: 44-52.
23. Spigel D, Jones S, Hainsworth J, Infante J, Greco FA, Thompson D, Doss H, and Burris H. A phase I trial to determine the safety of imatinib in combination with vatalanib in patients with advanced malignancies. Cancer Invest. 2011; 29: 308-312.
24. Weber DM, Graef T, Hussein M, Sobecks RM, Schiller GJ, Lupinacci L, Hardwick JS, and Jagannath S. Phase I trial of vorinostat combined with bortezomib for the treatment of relapsing and/or refractory multiple myeloma. Clin Lymphoma Myeloma Leuk. 2012; 12: 319-324.
25. Ciuleanu T, Tsai CM, Tsao CJ, Milanowski J, Amoroso D, Heo DS, Groen HJ, Szczesna A, Chung CY, Chao TY, Middleton G, Zeaiter A, Klingelschmitt G, et al. A phase II study of erlotinib in combination with bevacizumab versus chemotherapy plus bevacizumab in the first-line treatment of advanced non-squamous non-small cell lung cancer. Lung Cancer. 2013; 82: 276-281.
26. Cristofanilli M, Johnston SR, Manikhas A, Gomez HL, Gladkov O, Shao Z, Safina S, Blackwell KL, Alvarez RH, Rubin SD, Ranganathan S, Redhu S, and Trudeau ME. A randomized phase II study of lapatinib + pazopanib versus lapatinib in patients with HER2+ inflammatory breast cancer. Breast Cancer Res Treat. 2013; 137: 471-482.
27. Flaherty KT, Infante JR, Daud A, Gonzalez R, Kefford RF, Sosman J, Hamid O, Schuchter L, Cebon J, Ibrahim N, Kudchadkar R, Burris HA, 3rd, Falchook G, et al. Combined BRAF and MEK inhibition in melanoma with BRAF V600 mutations. N Engl J Med. 2012; 367: 1694-1703.
28. Groen HJ, Socinski MA, Grossi F, Juhasz E, Gridelli C, Baas P, Butts CA, Chmielowska E, Usari T, Selaru P, Harmon C, Williams JA, Gao F, et al. A randomized, double-blind, phase II study of erlotinib with or without sunitinib for the second-line treatment of metastatic non-small-cell lung cancer (NSCLC). Ann Oncol. 2013; 24: 2382-2389.
29. Slingluff CL, Jr., Petroni GR, Molhoek KR, Brautigan DL, Chianese-Bullock KA, Shada AL, Smolkin ME, Olson WC, Gaucher A, Chase CM, Grosh WW, Weiss GR, Wagenseller AG, et al. Clinical activity and safety of combination therapy with temsirolimus and bevacizumab for advanced melanoma: a phase II trial (CTEP 7190/Mel47). Clin Cancer Res. 2013; 19: 3611-3620.
30. Rini B, Szczylik C, Tannir NM, Koralewski P, Tomczak P, Deptala A, Dirix LY, Fishman M, Ramlau R, Ravaud A, Rogowski W, Kracht K, Sun YN, et al. AMG 386 in combination with sorafenib in patients with metastatic clear cell carcinoma of the kidney: a randomized, double-blind, placebo-controlled, phase 2 study. Cancer. 2012; 118: 6152-6161.
31. Grant BW, Jung SH, Johnson JL, Kostakoglu L, Hsi E, Byrd JC, Jones J, Leonard JP, Martin SE, and Cheson BD. A phase 2 trial of extended induction epratuzumab and rituximab for previously untreated follicular lymphoma: CALGB 50701. Cancer. 2013; 119: 3797-3804.
32. Clement-Duchene C, Natale RB, Jahan T, Krupitskaya Y, Osarogiagbon R, Sanborn RE, Bernstein ED, Dudek AZ, Latz JE, Shi P, and Wakelee HA. A phase II study of enzastaurin in combination with erlotinib in patients with previously treated advanced non-small cell lung cancer. Lung Cancer. 2012; 78: 57-62.
33. Schwartz GK, Tap WD, Qin LX, Livingston MB, Undevia SD, Chmielowski B, Agulnik M, Schuetze SM, Reed DR, Okuno SH, Ludwig JA, Keedy V, Rietschel P, et al. Cixutumumab and temsirolimus for patients with bone and soft-tissue sarcoma: a multicentre, open-label, phase 2 trial. Lancet Oncol. 2013; 14: 371-382.
34. Naing A, Lorusso P, Fu S, Hong D, Chen HX, Doyle LA, Phan AT, Habra MA, and Kurzrock R. Insulin growth factor receptor (IGF-1R) antibody cixutumumab combined with the mTOR inhibitor temsirolimus in patients with metastatic adrenocortical carcinoma. Br J Cancer. 2013; 108: 826-830.
35. Spigel DR, Ervin TJ, Ramlau RA, Daniel DB, Goldschmidt JH, Jr., Blumenschein GR, Jr., Krzakowski MJ, Robinet G, Godbert B, Barlesi F, Govindan R, Patel T, Orlov SV, et al. Randomized phase II trial of Onartuzumab in combination with erlotinib in patients with advanced non-small-cell lung cancer. J Clin Oncol. 2013; 31: 4105-4114.
36. Siena S, Van Cutsem E, Li M, Jungnelius U, Romano A, Beck R, Bencardino K, Elez ME, Prenen H, Sanchis M, Sartore-Bianchi A, Tejpar S, Gandhi A, et al. Phase II open-label study to assess efficacy and safety of lenalidomide in

combination with cetuximab in KRAS-mutant metastatic colorectal cancer. PLoS One. 2013; 8: e62264.

37. Lassen U, Sorensen M, Gaziel TB, Hasselbalch B, and Poulsen HS. Phase II study of bevacizumab and temsirolimus combination therapy for recurrent glioblastoma multiforme. Anticancer Res. 2013; 33: 1657-1660.
38. Peereboom DM, Ahluwalia MS, Ye X, Supko JG, Hilderbrand SL, Phuphanich S, Nabors LB, Rosenfeld MR, Mikkelsen T, Grossman SA, and New Approaches to Brain Tumor Therapy C. NABTT 0502: a phase II and pharmacokinetic study of erlotinib and sorafenib for patients with progressive or recurrent glioblastoma multiforme. Neuro Oncol. 2013; 15: 490-496.
39. Akerley W, Boucher K, Rich N, Egbert L, Harker G, Bylund J, Van Duren T, and Reddy C. A phase II study of bevacizumab and erlotinib as initial treatment for metastatic non-squamous, non-small cell lung cancer with serum proteomic evaluation. Lung Cancer. 2013; 79: 307-311.
40. Barnes JA, Jacobsen E, Feng Y, Freedman A, Hochberg EP, LaCasce AS, Armand P, Joyce R, Sohani AR, Rodig SJ, Neuberg D, Fisher DC, and Abramson JS. Everolimus in combination with rituximab induces complete responses in heavily pretreated diffuse large B-cell lymphoma. Haematologica. 2013; 98: 615-619.
41. Riggs H, Jalal SI, Baghdadi TA, Bhatia S, McClean J, Johnson C, Yu M, Taber D, Harb W, and Hanna N. Erlotinib and bevacizumab in newly diagnosed performance status 2 or elderly patients with nonsquamous non-small-cell lung cancer, a phase II study of the Hoosier Oncology Group: LUN04-77. Clin Lung Cancer. 2013; 14: 224-229.
42. Argiris A, Kotsakis AP, Hoang T, Worden FP, Savvides P, Gibson MK, Gyanchandani R, Blumenschein GR, Jr., Chen HX, Grandis JR, Harari PM, Kies MS, and Kim S. Cetuximab and bevacizumab: preclinical data and phase II trial in recurrent or metastatic squamous cell carcinoma of the head and neck. Ann Oncol. 2013; 24: 220-225.
43. Blumenschein GR, Jr., Ciuleanu T, Robert F, Groen HJ, Usari T, Ruiz-Garcia A, Tye L, Chao RC, and Juhasz E. Sunitinib plus erlotinib for the treatment of advanced/metastatic non-small-cell lung cancer: a lead-in study. J Thorac Oncol. 2012; 7: 1406-1416.
44. Govindarajan R, Siegel E, Makhoul I, and Williamson S. Bevacizumab and erlotinib in previously untreated inoperable and metastatic hepatocellular carcinoma. Am J Clin Oncol. 2013; 36: 254-257.
45. Hainsworth JD, Spigel DR, Burris HA, 3rd, Waterhouse D, Clark BL, and Whorf R. Phase II trial of bevacizumab and everolimus in patients with advanced renal cell carcinoma. J Clin Oncol. 2010; 28: 2131-2136.
46. Ko AH, Venook AP, Bergsland EK, Kelley RK, Korn WM, Dito E, Schillinger B, Scott J, Hwang J, and Tempero MA. A phase II study of bevacizumab plus erlotinib for gemcitabine-refractory metastatic pancreatic cancer. Cancer Chemother Pharmacol. 2010; 66: 1051-1057.
47. Altomare I, Bendell JC, Bullock KE, Uronis HE, Morse MA, Hsu SD, Zafar SY, Blobe GC, Pang H, Honeycutt W, Sutton L, and Hurwitz HI. A phase II trial of bevacizumab plus everolimus for patients with refractory metastatic colorectal cancer. Oncologist. 2011; 16: 1131-1137.
48. Kaseb AO, Garrett-Mayer E, Morris JS, Xiao L, Lin E, Onicescu G, Hassan MM, Hassabo HM, Iwasaki M, Deaton FL, Abbruzzese JL, and Thomas MB. Efficacy of bevacizumab plus erlotinib for advanced hepatocellular carcinoma and predictors of outcome: final results of a phase II trial. Oncology. 2012; 82: 67-74.
49. Hainsworth JD, Infante JR, Spigel DR, Peyton JD, Thompson DS, Lane CM, Clark BL, Rubin MS, Trent DF, and Burris HA, 3rd. Bevacizumab and everolimus in the treatment of patients with metastatic melanoma: a phase 2 trial of the Sarah Cannon Oncology Research Consortium. Cancer. 2010; 116: 4122-4129.
50. Reidy DL, Vakiani E, Fakih MG, Saif MW, Hecht JR, Goodman-Davis N, Hollywood E, Shia J, Schwartz J, Chandrawansa K, Dontabhaktuni A, Youssoufian H, Solit DB, et al. Randomized, phase II study of the insulin-like growth factor-1 receptor inhibitor IMC-A12, with or without cetuximab, in patients with cetuximab- or panitumumab-refractory metastatic colorectal cancer. J Clin Oncol. 2010; 28: 4240-4246.
51. Rohrberg KS, Olesen RK, Pfeiffer P, Ladekarl M, Pappot H, Christensen IJ, Hoyer-Hansen G, Sorensen M, Skov BG, Buysschaert I, Carmeliet P, and Lassen U. Phase II trial of erlotinib and bevacizumab in patients with advanced upper gastrointestinal cancers. Acta Oncol. 2012; 51: 234-242.
52. Lind JS, Dingemans AM, Groen HJ, Thunnissen FB, Bekers O, Heideman DA, Honeywell RJ, Giovannetti E, Peters GJ, Postmus PE, van Suylen RJ, and Smit EF. A multicenter phase II study of erlotinib and sorafenib in chemotherapy-naive patients with advanced non-small cell lung cancer. Clin Cancer Res. 2010; 16: 3078-3087.
53. Lonial S, Vij R, Harousseau JL, Facon T, Moreau P, Mazumder A, Kaufman JL, Leleu X, Tsao LC, Westland C, Singhal AK, and Jagannath S. Elotuzumab in combination with lenalidomide and low-dose dexamethasone in relapsed or refractory multiple myeloma. J Clin Oncol. 2012; 30: 1953-1959.
54. Ansell SM, Tang H, Kurtin PJ, Koenig PA, Inwards DJ, Shah K, Ziesmer SC, Feldman AL, Rao R, Gupta M, Erlichman C, and Witzig TE. Temsirolimus and rituximab in patients with relapsed or refractory mantle cell lymphoma: a phase 2 study. Lancet Oncol. 2011; 12: 361-368.
55. Spigel DR, Burris HA, 3rd, Greco FA, Shipley DL, Friedman EK, Waterhouse DM, Whorf RC, Mitchell RB, Daniel DB, Zangmeister J, Bass JD, and Hainsworth JD. Randomized, double-blind, placebo-controlled, phase II

trial of sorafenib and erlotinib or erlotinib alone in previously treated advanced non-small-cell lung cancer. J Clin Oncol. 2011; 29: 2582-2589.

56. Ko AH, Youssoufian H, Gurtler J, Dicke K, Kayaleh O, Lenz HJ, Keaton M, Katz T, Ballal S, and Rowinsky EK. A phase II randomized study of cetuximab and bevacizumab alone or in combination with gemcitabine as first-line therapy for metastatic pancreatic adenocarcinoma. Invest New Drugs. 2012; 30: 1597-1606.
57. Gridelli C, Morgillo F, Favaretto A, de Marinis F, Chella A, Cerea G, Mattioli R, Tortora G, Rossi A, Fasano M, Pasello G, Ricciardi S, Maione P, et al. Sorafenib in combination with erlotinib or with gemcitabine in elderly patients with advanced non-small-cell lung cancer: a randomized phase II study. Ann Oncol. 2011; 22: 1528-1534.
58. Sequist LV, von Pawel J, Garmey EG, Akerley WL, Brugger W, Ferrari D, Chen Y, Costa DB, Gerber DE, Orlov S, Ramlau R, Arthur S, Gorbachevsky I, et al. Randomized phase II study of erlotinib plus tivantinib versus erlotinib plus placebo in previously treated non-small-cell lung cancer. J Clin Oncol. 2011; 29: 3307-3315.
59. Ramalingam SS, Spigel DR, Chen D, Steins MB, Engelman JA, Schneider CP, Novello S, Eberhardt WE, Crino L, Habben K, Liu L, Janne PA, Brownstein CM, et al. Randomized phase II study of erlotinib in combination with placebo or R1507, a monoclonal antibody to insulin-like growth factor-1 receptor, for advanced-stage non-small-cell lung cancer. J Clin Oncol. 2011; 29: 4574-4580.
60. White D, Kassim A, Bhaskar B, Yi J, Wamstad K, and Paton VE. Results from AMBER, a randomized phase 2 study of bevacizumab and bortezomib versus bortezomib in relapsed or refractory multiple myeloma. Cancer. 2013; 119: 339-347.
61. Rini BI, Bellmunt J, Clancy J, Wang K, Niethammer AG, Hariharan S, and Escudier B. Randomized phase III trial of temsirolimus and bevacizumab versus interferon alfa and bevacizumab in metastatic renal cell carcinoma: INTORACT trial. J Clin Oncol. 2014; 32: 752-759.
62. Siu LL, Shapiro JD, Jonker DJ, Karapetis CS, Zalcberg JR, Simes J, Couture F, Moore MJ, Price TJ, Siddiqui J, Nott LM, Charpentier D, Liauw W, et al. Phase III randomized, placebo-controlled study of cetuximab plus brivanib alaninate versus cetuximab plus placebo in patients with metastatic, chemotherapy-refractory, wild-type K-RAS colorectal carcinoma: the NCIC Clinical Trials Group and AGITG CO.20 Trial. J Clin Oncol. 2013; 31: 2477-2484.
63. Scagliotti GV, Krzakowski M, Szczesna A, Strausz J, Makhson A, Reck M, Wierzbicki RF, Albert I, Thomas M, Miziara JE, Papai ZS, Karaseva N, Thongprasert S, et al. Sunitinib plus erlotinib versus placebo plus erlotinib in patients with previously treated advanced non-small-cell lung cancer: a phase III trial. J Clin Oncol. 2012; 30: 2070-2078.
64. Herbst RS, Ansari R, Bustin F, Flynn P, Hart L, Otterson GA, Vlahovic G, Soh CH, O'Connor P, and Hainsworth J. Efficacy of bevacizumab plus erlotinib versus erlotinib alone in advanced non-small-cell lung cancer after failure of standard first-line chemotherapy (BeTa): a double-blind, placebo-controlled, phase 3 trial. Lancet. 2011; 377: 1846-1854.
65. Fayad L, Offner F, Smith MR, Verhoef G, Johnson P, Kaufman JL, Rohatiner A, Advani A, Foran J, Hess G, Coiffier B, Czuczman M, Gine E, et al. Safety and clinical activity of a combination therapy comprising two antibody-based targeting agents for the treatment of non-Hodgkin lymphoma: results of a phase I/II study evaluating the immunoconjugate inotuzumab ozogamicin with rituximab. J Clin Oncol. 2013; 31: 573-583.
66. Lee EQ, Kuhn J, Lamborn KR, Abrey L, DeAngelis LM, Lieberman F, Robins HI, Chang SM, Yung WK, Drappatz J, Mehta MP, Levin VA, Aldape K, et al. Phase I/II study of sorafenib in combination with temsirolimus for recurrent glioblastoma or gliosarcoma: North American Brain Tumor Consortium study 05-02. Neuro Oncol. 2012; 14: 1511-1518.
67. Haura EB, Tanvetyanon T, Chiappori A, Williams C, Simon G, Antonia S, Gray J, Litschauer S, Tetteh L, Neuger A, Song L, Rawal B, Schell MJ, et al. Phase I/II study of the Src inhibitor dasatinib in combination with erlotinib in advanced non-small-cell lung cancer. J Clin Oncol. 2010; 28: 1387-1394.
68. Larkin JM, Ferguson TR, Pickering LM, Edmonds K, James MG, Thomas K, Banerji U, Berns B, de Boer C, and Gore ME. A phase I/II trial of sorafenib and infliximab in advanced renal cell carcinoma. Br J Cancer. 2010; 103: 1149-1153.
69. Morrow PK, Wulf GM, Ensor J, Booser DJ, Moore JA, Flores PR, Xiong Y, Zhang S, Krop IE, Winer EP, Kindelberger DW, Coviello J, Sahin AA, et al. Phase I/II study of trastuzumab in combination with everolimus (RAD001) in patients with HER2-overexpressing metastatic breast cancer who progressed on trastuzumab-based therapy. J Clin Oncol. 2011; 29: 3126-3132.
70. Weickhardt A, Doebele R, Oton A, Lettieri J, Maxson D, Reynolds M, Brown A, Jackson MK, Dy G, Adjei A, Fetterly G, Lu X, Franklin W, et al. A phase I/II study of erlotinib in combination with the anti-insulin-like growth factor-1 receptor monoclonal antibody IMC-A12 (cixutumumab) in patients with advanced non-small cell lung cancer. J Thorac Oncol. 2012; 7: 419-426.
71. Padda SK, Krupitskaya Y, Chhatwani L, Fisher GA, Colevas AD, San Pedro-Salcedo M, Decker R, Latz JE, and Wakelee HA. A phase I dose-escalation and pharmacokinetic study of enzastaurin and erlotinib in patients with advanced solid tumors. Cancer Chemother Pharmacol. 2012; 69: 1013-1020.

72. Monk BJ, Mas Lopez L, Zarba JJ, Oaknin A, Tarpin C, Termrungruanglert W, Alber JA, Ding J, Stutts MW, and Pandite LN. Phase II, open-label study of pazopanib or lapatinib monotherapy compared with pazopanib plus lapatinib combination therapy in patients with advanced and recurrent cervical cancer. J Clin Oncol. 2010; 28: 3562-3569.
73. Simonelli M, Zucali PA, Lorenzi E, Rubino L, De Vincenzo F, De Sanctis R, Perrino M, Mancini L, Di Tommaso L, Rimassa L, Masci G, Zuradelli M, Suter MB, et al. Phase I pharmacokinetic and pharmacodynamic study of lapatinib in combination with sorafenib in patients with advanced refractory solid tumors. Eur J Cancer. 2013; 49: 989-998.
74. Langenberg MH, Witteveen PO, Lankheet NA, Roodhart JM, Rosing H, van den Heuvel IJ, Beijnen JH, and Voest EE. Phase 1 study of combination treatment with PTK 787/ZK 222584 and cetuximab for patients with advanced solid tumors: safety, pharmacokinetics, pharmacodynamics analysis. Neoplasia. 2010; 12: 206-213.
75. Rugo HS, Chien AJ, Franco SX, Stopeck AT, Glencer A, Lahiri S, Arbushites MC, Scott J, Park JW, Hudis C, Nulsen B, and Dickler MN. A phase II study of lapatinib and bevacizumab as treatment for HER2-overexpressing metastatic breast cancer. Breast Cancer Res Treat. 2012; 134: 13-20.
76. Reardon DA, Desjardins A, Vredenburgh JJ, Gururangan S, Friedman AH, Herndon JE, 2nd, Marcello J, Norfleet JA, McLendon RE, Sampson JH, and Friedman HS. Phase 2 trial of erlotinib plus sirolimus in adults with recurrent glioblastoma. J Neurooncol. 2010; 96: 219-230.
77. Ganesan P, Piha-Paul S, Naing A, Falchook G, Wheler J, Janku F, Zinner R, Laday S, Kies M, and Tsimberidou AM. Phase I clinical trial of lenalidomide in combination with temsirolimus in patients with advanced cancer. Invest New Drugs. 2013; 31: 1505-1513.
78. Holkova B, Supko JG, Ames MM, Reid JM, Shapiro GI, Perkins EB, Ramakrishnan V, Tombes MB, Honeycutt C, McGovern RM, Kmieciak M, Shrader E, Wellons MD, et al. A phase I trial of vorinostat and alvocidib in patients with relapsed, refractory, or poor prognosis acute leukemia, or refractory anemia with excess blasts-2. Clin Cancer Res. 2013; 19: 1873-1883.
79. Wang M, Fayad L, Wagner-Bartak N, Zhang L, Hagemeister F, Neelapu SS, Samaniego F, McLaughlin P, Fanale M, Younes A, Cabanillas F, Fowler N, Newberry KJ, et al. Lenalidomide in combination with rituximab for patients with relapsed or refractory mantle-cell lymphoma: a phase 1/2 clinical trial. Lancet Oncol. 2012; 13: 716-723.
80. Dy GK, Infante JR, Eckhardt SG, Novello S, Ma WW, Jones SF, Huff A, Wang Q, Suttle AB, Ottesen LH, Adjei AA, and Burris HA, 3rd. Phase Ib trial of the oral angiogenesis inhibitor pazopanib administered concurrently with erlotinib. Invest New Drugs. 2013; 31: 891-899.
81. Molina AM, Hutson TE, Larkin J, Gold AM, Wood K, Carter D, Motzer R, and Michaelson MD. A phase 1b clinical trial of the multi-targeted tyrosine kinase inhibitor lenvatinib (E7080) in combination with everolimus for treatment of metastatic renal cell carcinoma (RCC). Cancer Chemother Pharmacol. 2014; 73: 181-189.
82. Davies MA, Fox PS, Papadopoulos NE, Bedikian AY, Hwu WJ, Lazar AJ, Prieto VG, Culotta KS, Madden TL, Xu Q, Huang S, Deng W, Ng CS, et al. Phase I study of the combination of sorafenib and temsirolimus in patients with metastatic melanoma. Clin Cancer Res. 2012; 18: 1120-1128.
83. Motzer RJ, Hudes GR, Ginsberg MS, Baum MS, Harmon CS, Kim ST, Chen I, and Redman BG. Phase I/II trial of sunitinib plus gefitinib in patients with metastatic renal cell carcinoma. Am J Clin Oncol. 2010; 33: 614-618.
84. Ghobrial IM, Weller E, Vij R, Munshi NC, Banwait R, Bagshaw M, Schlossman R, Leduc R, Chuma S, Kunsman J, Laubach J, Jakubowiak AJ, Maiso P, et al. Weekly bortezomib in combination with temsirolimus in relapsed or relapsed and refractory multiple myeloma: a multicentre, phase 1/2, open-label, dose-escalation study. Lancet Oncol. 2011; 12: 263-272.
85. Bose P, Perkins EB, Honeycut C, Wellons MD, Stefan T, Jacobberger JW, Kontopodis E, Beumer JH, Egorin MJ, Imamura CK, Douglas Figg W, Sr., Karp JE, Koc ON, et al. Phase I trial of the combination of flavopiridol and imatinib mesylate in patients with Bcr-Abl+ hematological malignancies. Cancer Chemother Pharmacol. 2012; 69: 1657-1667.
86. Ghobrial IM, Xie W, Padmanabhan S, Badros A, Rourke M, Leduc R, Chuma S, Kunsman J, Warren D, Poon T, Harris B, Sam A, Anderson KC, et al. Phase II trial of weekly bortezomib in combination with rituximab in untreated patients with Waldenstrom Macroglobulinemia. Am J Hematol. 2010; 85: 670-674.
87. Ghobrial IM, Hong F, Padmanabhan S, Badros A, Rourke M, Leduc R, Chuma S, Kunsman J, Warren D, Harris B, Sam A, Anderson KC, Richardson PG, et al. Phase II trial of weekly bortezomib in combination with rituximab in relapsed or relapsed and refractory Waldenstrom macroglobulinemia. J Clin Oncol. 2010; 28: 1422-1428.
88. Trarbach T, Schultheis B, Gauler TC, Schneider V, Strumberg D, Eberhardt WE, Le Scouiller S, Marotti M, Brown KH, and Drevs J. Phase I open-label study of cediranib, an oral inhibitor of VEGF signalling, in combination with the oral Src inhibitor saracatinib in patients with advanced solid tumours. Invest New Drugs. 2012; 30: 1962-1971.
89. Kotasek D, Tebbutt N, Desai J, Welch S, Siu LL, McCoy S, Sun YN, Johnson J, Adewoye AH, and Price T. Safety and pharmacokinetics of motesanib in combination with

gemcitabine and erlotinib for the treatment of solid tumors: a phase 1b study. BMC Cancer. 2011; 11: 313.

90. Wolpin BM, Ng K, Zhu AX, Abrams T, Enzinger PC, McCleary NJ, Schrag D, Kwak EL, Allen JN, Bhargava P, Chan JA, Goessling W, Blaszkowsky LS, et al. Multicenter phase II study of tivozanib (AV-951) and everolimus (RAD001) for patients with refractory, metastatic colorectal cancer. Oncologist. 2013; 18: 377-378.
91. Weickhardt AJ, Price TJ, Chong G, Gebski V, Pavlakis N, Johns TG, Azad A, Skrinos E, Fluck K, Dobrovic A, Salemi R, Scott AM, Mariadason JM, et al. Dual targeting of the epidermal growth factor receptor using the combination of cetuximab and erlotinib: preclinical evaluation and results of the phase II DUX study in chemotherapy-refractory, advanced colorectal cancer. J Clin Oncol. 2012; 30: 1505-1512.
92. Dimopoulos M, Siegel DS, Lonial S, Qi J, Hajek R, Facon T, Rosinol L, Williams C, Blacklock H, Goldschmidt H, Hungria V, Spencer A, Palumbo A, et al. Vorinostat or placebo in combination with bortezomib in patients with multiple myeloma (VANTAGE 088): a multicentre, randomised, double-blind study. Lancet Oncol. 2013; 14: 1129-1140.
93. Blackwell KL, Burstein HJ, Storniolo AM, Rugo HS, Sledge G, Aktan G, Ellis C, Florance A, Vukelja S, Bischoff J, Baselga J, and O'Shaughnessy J. Overall survival benefit with lapatinib in combination with trastuzumab for patients with human epidermal growth factor receptor 2-positive metastatic breast cancer: final results from the EGF104900 Study. J Clin Oncol. 2012; 30: 2585-2592.
94. Janjigian YY, Azzoli CG, Krug LM, Pereira LK, Rizvi NA, Pietanza MC, Kris MG, Ginsberg MS, Pao W, Miller VA, and Riely GJ. Phase I/II trial of cetuximab and erlotinib in patients with lung adenocarcinoma and acquired resistance to erlotinib. Clin Cancer Res. 2011; 17: 2521-2527.
95. Friday BB, Anderson SK, Buckner J, Yu C, Giannini C, Geoffroy F, Schwerkoske J, Mazurczak M, Gross H, Pajon E, Jaeckle K, and Galanis E. Phase II trial of vorinostat in combination with bortezomib in recurrent glioblastoma: a north central cancer treatment group study. Neuro Oncol. 2012; 14: 215-221.
96. Schmidinger M, Szczylik C, Sternberg CN, Kania M, Kelly CS, Decker R, Hamid O, Faelker T, and Escudier B. Dose escalation and pharmacokinetics study of enzastaurin and sunitinib versus placebo and sunitinib in patients with metastatic renal cell carcinoma. Am J Clin Oncol. 2012; 35: 493-497.
97. Gandhi L, Bahleda R, Tolaney SM, Kwak EL, Cleary JM, Pandya SS, Hollebecque A, Abbas R, Ananthakrishnan R, Berkenblit A, Krygowski M, Liang Y, Turnbull KW, et al. Phase I study of neratinib in combination with temsirolimus in patients with human epidermal growth factor receptor 2-dependent and other solid tumors. J Clin Oncol. 2014; 32: 68-75.
98. Bauman JE, Arias-Pulido H, Lee SJ, Fekrazad MH, Ozawa H, Fertig E, Howard J, Bishop J, Wang H, Olson GT, Spafford MJ, Jones DV, and Chung CH. A phase II study of temsirolimus and erlotinib in patients with recurrent and/or metastatic, platinum-refractory head and neck squamous cell carcinoma. Oral Oncol. 2013; 49: 461-467.
99. Modi S, Saura C, Henderson C, Lin NU, Mahtani R, Goddard J, Rodenas E, Hudis C, O'Shaughnessy J, and Baselga J. A multicenter trial evaluating retaspimycin HCL (IPI-504) plus trastuzumab in patients with advanced or metastatic HER2-positive breast cancer. Breast Cancer Res Treat. 2013; 139: 107-113.
100. Baselga J, Bradbury I, Eidtmann H, Di Cosimo S, de Azambuja E, Aura C, Gomez H, Dinh P, Fauria K, Van Dooren V, Aktan G, Goldhirsch A, Chang TW, et al. Lapatinib with trastuzumab for HER2-positive early breast cancer (NeoALTTO): a randomised, open-label, multicentre, phase 3 trial. Lancet. 2012; 379: 633-640.
101. Holkova B, Perkins EB, Ramakrishnan V, Tombes MB, Shrader E, Talreja N, Wellons MD, Hogan KT, Roodman GD, Coppola D, Kang L, Dawson J, Stuart RK, et al. Phase I trial of bortezomib (PS-341; NSC 681239) and alvocidib (flavopiridol; NSC 649890) in patients with recurrent or refractory B-cell neoplasms. Clin Cancer Res. 2011; 17: 3388-3397.
102. Badoux XC, Keating MJ, Wen S, Wierda WG, O'Brien SM, Faderl S, Sargent R, Burger JA, and Ferrajoli A. Phase II study of lenalidomide and rituximab as salvage therapy for patients with relapsed or refractory chronic lymphocytic leukemia. J Clin Oncol. 2013; 31: 584-591.
103. Chan JA, Mayer RJ, Jackson N, Malinowski P, Regan E, and Kulke MH. Phase I study of sorafenib in combination with everolimus (RAD001) in patients with advanced neuroendocrine tumors. Cancer Chemother Pharmacol. 2013; 71: 1241-1246.
104. Ganesan P, Piha-Paul S, Naing A, Falchook G, Wheler J, Fu S, Hong DS, Kurzrock R, Janku F, Laday S, Bedikian AY, Kies M, Wolff RA, et al. Phase I clinical trial of lenalidomide in combination with sorafenib in patients with advanced cancer. Invest New Drugs. 2014; 32: 279-286.
105. Papadimitrakopoulou VA, Soria JC, Jappe A, Jehl V, Klimovsky J, and Johnson BE. Everolimus and erlotinib as second- or third-line therapy in patients with advanced non-small-cell lung cancer. J Thorac Oncol. 2012; 7: 1594-1601.
106. Harzstark AL, Small EJ, Weinberg VK, Sun J, Ryan CJ, Lin AM, Fong L, Brocks DR, and Rosenberg JE. A phase 1 study of everolimus and sorafenib for metastatic clear cell renal cell carcinoma. Cancer. 2011; 117: 4194-4200.
107. Oki Y, Buglio D, Fanale M, Fayad L, Copeland A, Romaguera J, Kwak LW, Pro B, de Castro Faria S, Neelapu S, Fowler N, Hagemeister F, Zhang J, et al. Phase I study of panobinostat plus everolimus in patients with relapsed or refractory lymphoma. Clin Cancer Res. 2013; 19: 6882-6890.

108. Besse B, Leighl N, Bennouna J, Papadimitrakopoulou VA, Blais N, Traynor AM, Soria JC, Gogov S, Miller N, Jehl V, and Johnson BE. Phase II study of everolimus-erlotinib in previously treated patients with advanced non-small-cell lung cancer. Ann Oncol. 2014; 25: 409-415.

109. Price KA, Azzoli CG, Krug LM, Pietanza MC, Rizvi NA, Pao W, Kris MG, Riely GJ, Heelan RT, Arcila ME, and Miller VA. Phase II trial of gefitinib and everolimus in advanced non-small cell lung cancer. J Thorac Oncol. 2010; 5: 1623-1629.

110. Macpherson IR, Poondru S, Simon GR, Gedrich R, Brock K, Hopkins CA, Stewart K, Stephens A, and Evans TR. A phase 1 study of OSI-930 in combination with erlotinib in patients with advanced solid tumours. Eur J Cancer. 2013; 49: 782-789.

111. Harvey RD, Owonikoko TK, Lewis CM, Akintayo A, Chen Z, Tighiouart M, Ramalingam SS, Fanucchi MP, Nadella P, Rogatko A, Shin DM, El-Rayes B, Khuri FR, et al. A phase 1 Bayesian dose selection study of bortezomib and sunitinib in patients with refractory solid tumor malignancies. Br J Cancer. 2013; 108: 762-765.

112. Wilky BA, Rudek MA, Ahmed S, Laheru DA, Cosgrove D, Donehower RC, Nelkin B, Ball D, Doyle LA, Chen H, Ye X, Bigley G, Womack C, et al. A phase I trial of vertical inhibition of IGF signalling using cixutumumab, an anti-IGF-1R antibody, and selumetinib, an MEK 1/2 inhibitor, in advanced solid tumours. Br J Cancer. 2015; 112: 24-31.

113. Gadgeel SM, Lew DL, Synold TW, LoRusso P, Chung V, Christensen SD, Smith DC, Kingsbury L, Hoering A, and Kurzrock R. Phase I study evaluating the combination of lapatinib (a Her2/Neu and EGFR inhibitor) and everolimus (an mTOR inhibitor) in patients with advanced cancers: South West Oncology Group (SWOG) Study S0528. Cancer Chemother Pharmacol. 2013; 72: 1089-1096.

114. Bitting RL, Healy P, Creel PA, Turnbull J, Morris K, Wood SY, Hurwitz HI, Starr MD, Nixon AB, Armstrong AJ, and George DJ. A phase Ib study of combined VEGFR and mTOR inhibition with vatalanib and everolimus in patients with advanced renal cell carcinoma. Clin Genitourin Cancer. 2014; 12: 241-250.

115. Kumar SK, Jett J, Marks R, Richardson R, Quevedo F, Moynihan T, Croghan G, Markovic SN, Bible KC, Qin R, Tan A, Molina J, Kaufmann SH, et al. Phase 1 study of sorafenib in combination with bortezomib in patients with advanced malignancies. Invest New Drugs. 2013; 31: 1201-1206.

116. Ma CX, Suman VJ, Goetz M, Haluska P, Moynihan T, Nanda R, Olopade O, Pluard T, Guo Z, Chen HX, Erlichman C, Ellis MJ, and Fleming GF. A phase I trial of the IGF-1R antibody Cixutumumab in combination with temsirolimus in patients with metastatic breast cancer. Breast Cancer Res Treat. 2013; 139: 145-153.

117. Liu JF, Tolaney SM, Birrer M, Fleming GF, Buss MK, Dahlberg SE, Lee H, Whalen C, Tyburski K, Winer E, Ivy P, and Matulonis UA. A Phase 1 trial of the poly(ADP-ribose) polymerase inhibitor olaparib (AZD2281) in combination with the anti-angiogenic cediranib (AZD2171) in recurrent epithelial ovarian or triple-negative breast cancer. Eur J Cancer. 2013; 49: 2972-2978.

118. Waqar SN, Gopalan PK, Williams K, Devarakonda S, and Govindan R. A phase I trial of sunitinib and rapamycin in patients with advanced non-small cell lung cancer. Chemotherapy. 2013; 59: 8-13.

119. Johnston SR, Gomez H, Stemmer SM, Richie M, Durante M, Pandite L, Goodman V, and Slamon D. A randomized and open-label trial evaluating the addition of pazopanib to lapatinib as first-line therapy in patients with HER2-positive advanced breast cancer. Breast Cancer Res Treat. 2013; 137: 755-766.

120. Dickson MA, Rathkopf DE, Carvajal RD, Grant S, Roberts JD, Reid JM, Ames MM, McGovern RM, Lefkowitz RA, Gonen M, Cane LM, Dials HJ, and Schwartz GK. A phase I pharmacokinetic study of pulse-dose vorinostat with flavopiridol in solid tumors. Invest New Drugs. 2011; 29: 1004-1012.

Supplementary Table S1: Phase I-III Clinical Trials of Two Targeted Agents (N = 144 Trials)

See Supplementary File 1
