# Supplementary material for: Dosing de novo combinations of two targeted drugs: Towards a customized precision medicine approach to advanced cancers
Source: Oncotarget. 2016 Jan 25;7(10):11310–20. doi: 10.18632/oncotarget.7023 (PMC4905475; doi:10.18632/oncotarget.7023)
Supplement: Supplementary file 2 [file oncotarget-07-11310-s002.doc]

**Supplemental Table 1: Phase I-III Clinical Trials of Two Targeted Agents (N = 144 Trials)**

| **Targeted Agent 1** | **Targeted Agent 2** | **Disease** | **Phase** | **Number of Patients on Combination** |
| --- | --- | --- | --- | --- |
| ABT-510 | bevacizumab | solid tumors | 1 | 34 |
| alemtuzumab | rituximab | chronic lymphocytic leukemia/small lymphocytic lymphoma | 1 | 28 |
| alvespimycin HCL | trastuzumab | solid tumors | 1 | 28 |
| alvocidib | bortezomib | B-cell neoplasms | 1 | 16 |
| alvocidib | vorinostat | acute leukemia | 1 | 28 |
| alvocidib | vorinostat | solid tumors | 1 | 34 |
| bevacizumab | olaparib | advanced solid tumors | 1 | 12 |
| bevacizumab | panobinostat | gliomas | 1 | 12 |
| bevacizumab | rapamycin | hepatocellular carcinoma | 1 | 24 |
| bevacizumab | rilotumumab | advanced solid tumors | 1 | 14 |
| bevacizumab | sirolimus | advanced malignancies | 1 | 28 |
| bevacizumab | telatinib | advanced solid tumors | 1 | 26 |
| bevacizumab | vandetanib | solid tumors and lymphomas | 1 | 15 |
| bevacizumab | vatalinib | advanced malignancies | 1 | 27 |
| bevacizumab | ridaforolimus | advanced cancers | 1 | 17 |
| bortezomib | sorafenib | advanced malignancies | 1 | 14 |
| bortezomib | sunitinib | refractory solid tumors | 1 | 31 |
| bortezomib | vorinostat | multiple myeloma | 1 | 34 |
| brivanib alaninate | cetuximab | gastrointestinal malignancies | 1 | 62 |
| cediranib | gefitinib | advanced tumors | 1 | 90 |
| cediranib | olaparib | recurrent epithelial ovarian or triple negative breast cancer | 1 | 28 |
| cediranib | sarcatinib | advanced solid tumors | 1 | 39 |
| cetuximab | dasatinib | advanced solid malignancies | 1 | 31 |
| cetuximab | everolimus | solid tumors | 1 | 29 |
| cetuximab | vatalanib | advanced solid tumors | 1 | 16 |
| cixutumumab | selumetinib | advanced solid tumors | 1 | 30 |
| cixutumumab | temsirolimus | breast cancer | 1 | 26 |
| enzastaurin | erlotinib | advanced solid tumors | 1 | 16 |
| erlotinib | everolimus | non-small cell lung cancer | 1 | 94 |
| erlotinib | motesanib | solid tumors | 1 | 56 |
| erlotinib | OSI-930 | solid tumors | 1 | 20 |
| erlotinib | pazopanib | solid tumors | 1 | 33 |
| erlotinib | pertuzumab | non-small cell lung cancer | 1 | 15 |
| erlotinib | sirolimus | malignant glioma | 1 | 19 |
| erlotinib | sorafenib | solid tumors | 1 | 11 |
| erlotinib | sunitinib | non-small cell lung cancer | 1 | 11 |
| erlotinib | tivantinib | solid tumors | 1 | 32 |
| erlotinib | tivantinib | non-small cell lung cancer | 1 | 25 |
| everolimus | figitumumab | sarcomas and solid tumors | 1 | 21 |
| everolimus | lapatinib | advanced solid tumors | 1 | 77 |
| everolimus | lenvatinib | renal cell carcinoma | 1 | 20 |
| everolimus | panobinostat | relapsed or refractory Hodgkin and non-Hodgkin lymphoma | 1 | 30 |
| everolimus | sorafenib | metastatic clear cell renal cell carcinoma | 1 | 20 |
| everolimus | sorafenib | advanced hepatocellular carcinoma | 1 | 30 |
| everolimus | sorafenib | neuroendocrine tumors | 1 | 21 |
| everolimus | sunitinib | metastatic renal cell carcinoma | 1 | 20 |
| everolimus | vatalanib | metastatic renal cell carcinoma | 1 | 32 |
| flavopiridol | imatinib | hematologic malignancies (BCR-ABL positive) | 1 | 21 |
| gefitinib | nimotuzumab | non-small cell lung cancer | 1 | 16 |
| imatinib | vatalanib | advanced malignancies | 1 | 45 |
| lapatinib | pazopanib | solid tumors | 1 | 75 |
| lapatinib | sorafenib | solid tumors | 1 | 30 |
| lenalidomide | sorafenib | advanced malignancies | 1 | 41 |
| lenalidomide | temsirolimus | advanced solid tumors | 1 | 43 |
| MK2206 | trastuzumab | Her2/neu positive solid tumors | 1 | 31 |
| motesanib | panitumumab | non-small cell lung cancer | 1 | 16 |
| neratinib | temsirolimus | Her2/neu positive solid tumors | 1 | 60 |
| pazopanib | temsirolimus | advanced solid tumors | 1 | 8 |
| rapamycin | sunitinib | advanced non-small cell lung cancer | 1 | 19 |
| sirolimus | sorafenib | advanced solid cancers | 1 | 19 |
| sorafenib | tanespimycin | solid tumors | 1 | 27 |
| sorafenib | temserolimus | melanoma | 1 | 25 |
| sorafenib | temsirolimus | hepatocellular carcinoma | 1 | 25 |
| temsirolimus | tivozanib | renal cell carcinoma | 1 | 27 |
| bortezomib | temsirolimus | multiple myeloma | 1 and 2 | 63 |
| cetuximab | erlotinib | lung adenocarcinoma | 1 and 2 | 19 |
| cixutumumab | erlotinib | non-small cell lung cancer | 1 and 2 | 18 |
| dasatinib | erlotinib | non-small cell lung cancer | 1 and 2 | 34 |
| everolimus | imatinib | gastrointestinal stromal tumor | 1 and 2 | 117 |
| everolimus | trastuzumab | metastatic breast cancer | 1 and 2 | 47 |
| gefitinib | sunitinib | metastatic renal cell carcinoma | 1 and 2 | 42 |
| infliximab | sorafenib | renal cell carcinoma | 1 and 2 | 16 |
| inotuzumab ozogamicin | rituximab | relapsed/refractory CD20 positive/CD22 positive B-cell non-Hodgkin lymphoma | 1 and 2 | 118 |
| lenalidomide | rituximab | mantle cell lymphoma | 1 and 2 | 52 |
| sorafenib | temsirolimus | recurrent glioblastoma multiforme or gliosarcoma | 1 and 2 | 31 |
| AMG386 | sorafenib | clear cell kidney carcinoma | 2 | 152 |
| bevacizumab | bortezomib | multiple myeloma | 2 | 102 |
| bevacizumab | cetuximab | squamous cell carcinoma of the head and neck | 2 | 46 |
| bevacizumab | cetuximab | pancreatic cancer | 2 | 31 |
| bevacizumab | erlotinib | metastatic pancreatic cancer | 2 | 36 |
| bevacizumab | erlotinib | advanced hepatocellular carcinoma | 2 | 59 |
| bevacizumab | erlotinib | non-squamous non-small cell lung cancer | 2 | 50 |
| bevacizumab | erlotinib | hepatocellular carcinoma | 2 | 21 |
| bevacizumab | erlotinib | biliary cancer | 2 | 53 |
| bevacizumab | erlotinib | advanced hepatocellular carcinoma | 2 | 51 |
| bevacizumab | erlotinib | upper gastrointestinal cancers | 2 | 102 |
| bevacizumab | erlotinib | non-small cell lung cancer | 2 | 63 |
| bevacizumab | erlotinib | non-squamous non-small cell lung cancer | 2 | 25 |
| bevacizumab | everolimus | advanced renal cell carcinoma | 2 | 80 |
| bevacizumab | everolimus | metastatic colorectal cancer | 2 | 50 |
| bevacizumab | everolimus | metastatic melanoma | 2 | 57 |
| bevacizumab | everolimus | metastatic renal cell carcinoma | 2 | 10 |
| bevacizumab | lapatinib | metastatic breast cancer | 2 | 52 |
| bevacizumab | sorafenib | recurrent glioblastoma multiforme | 2 | 54 |
| bevacizumab | sorafenib | metastatic breast cancer | 2 | 18 |
| bevacizumab | sorafenib | advanced neuroendocrine tumors | 2 | 44 |
| bevacizumab | temsirolimus | glioblastoma multiforme | 2 | 13 |
| bevacizumab | temsirolimus | endometrial cancer | 2 | 49 |
| bevacizumab | temsirolimus | advanced melanoma | 2 | 17 |
| bortezomib | rituximab | Waldenstrom macroglobunemia | 2 | 26 |
| bortezomib | rituximab | Waldenstrom macroglobulinemia | 2 | 37 |
| bortezomib | vorinostat | glioblastoma multiforme | 2 | 37 |
| cetuximab | cixutumumab | metastatic colon cancer | 2 | 41 |
| cetuximab | erlotinib | advanced colorectal cancer | 2 | 50 |
| cetuximab | lenalidomide | colorectal cancer (Kras mutant) | 2 | 51 |
| cixutumumab | temsirolimus | bone and soft tissue sarcoma | 2 | 174 |
| cixutumumab | temsirolimus | metastatic adrenocortical carcinoma | 2 | 26 |
| dabrafenib | trametinib | melanoma (BRAF V600 mutated) | 2 | 247 |
| elotuzumab | lenalidomide | multiple myeloma | 2 | 29 |
| enzastaurin | erlotinib | non-small cell lung cancer | 2 | 49 |
| enzastaurin | sunitinib | renal cell carcinoma | 2 | 17 |
| epratuzumab | rituximab | follicular lymphoma | 2 | 59 |
| erlotinib | everolimus | non-small cell lung cancer | 2 | 66 |
| erlotinib | onartuzumab | non-small cell lung cancer | 2 | 69 |
| erlotinib | R1507 | non-small cell lung cancer | 2 | 114 |
| erlotinib | sirolimus | glioblastoma multiforme | 2 | 32 |
| erlotinib | sorafenib | non-small cell lung cancer | 2 | 50 |
| erlotinib | sorafenib | recurrent glioblastoma multiforme | 2 | 51 |
| erlotinib | sorafenib | non-small cell lung cancer | 2 | 29 |
| erlotinib | sorafenib | non-small cell lung cancer | 2 | 111 |
| erlotinib | sunitinib | metastatic non-small cell lung cancer | 2 | 64 |
| erlotinib | sunitinib | non-small cell lung cancer | 2 | 30 |
| erlotinib | temsirolimus | squamous cell carcinoma of the head and neck | 2 | 12 |
| erlotinib | tivantinib | non-small cell lung cancer | 2 | 84 |
| everolimus | gefitinib | non-small cell lung cancer | 2 | 62 |
| everolimus | imatinib | renal cell carcinoma | 2 | 19 |
| everolimus | rituximab | diffuse large B cell lymphoma | 2 | 24 |
| everolimus | tivozanib | colorectal cancer | 2 | 40 |
| lapatinib | pazopanib | Her2/neu positive inflammatory breast cancer | 2 | 164 |
| lapatinib | pazopanib | cervical cancer | 2 | 76 |
| lapatinib | pazopanib | Her2/neu positive breast cancer | 2 | 190 |
| lenalidomide | rituximab | relapsed or refractory CLL | 2 | 59 |
| pertuzumab | trastuzumab | metastatic breast cancer | 2 | 66 |
| pertuzumab | trastuzumab | Her2/neu positive breast cancer | 2 | 107 |
| retaspimycin HCL | trastuzumab | Her2/neu positive breast cancer | 2 | 26 |
| rituximab | temsirolimus | mantle cell lymphoma | 2 | 69 |
| bevacizumab | erlotinib | non-small cell lung cancer | 3 | 319 |
| bevacizumab | erlotinib | metastatic colorectal cancer | 3 | 80 |
| bevacizumab | temsirolimus | renal cell carcinoma | 3 | 400 |
| bortezomib | vorinostat | multiple myeloma | 3 | 317 |
| brivanib alaninate | cetuximab | metastatic colorectal cancer (Kras wild type) | 3 | 376 |
| erlotinib | sunitinib | non-small cell lung cancer | 3 | 480 |
| lapatinib | trastuzumab | Her2/neu positive breast cancer | 3 | 291 |
| lapatinib | trastuzumab | Her2/neu positive breast cancer | 3 | 152 |
